# Budget impact analysis of subcutaneous infliximab (CT-P13 SC) for treating inflammatory bowel disease in Saudi Arabia: Analysis from payer perspective

**DOI:** 10.1371/journal.pone.0312603

**Published:** 2024-11-12

**Authors:** Nimer S. Alkhatib, Abdulaali R. Almutairi, Majid Almadi, Shiraz Halloush, Yazed Sulaiman H. Al-Ruthia, Omar Rashdan, Samah Al-Shatnawi, Nahla A. Azzam, Mahmoud H. Mosli, Amal M. Badawoud, Majed S. Al Yami, Abdulaziz Alhossan, Ibtisam AlHarbi

**Affiliations:** 1 Faculty of Pharmacy, Al-Zaytoonah University of Jordan, Amman, Jordan; 2 Path Economics, LLC, Amman, Jordan; 3 Drug Sector, Saudi Food and Drug Authority, Riyadh, Saudi Arabia; 4 Gastroenterology Division, Department of Medicine, King Saud University Medical City, King Saud University, Riyadh, Saudi Arabia; 5 Division of Gastroenterology, The McGill University Health Center, Montreal General Hospital, McGill University, Montreal, Canada; 6 Faculty of Pharmacy, Applied Science Private University, Amman, Jordan; 7 Department of Clinical Pharmacy, College of Pharmacy, King Saud University, Riyadh, Saudi Arabia; 8 College of Pharmacy, Middle East University, Amman, Jordan; 9 Faculty of Pharmacy, Jordan University of Science and Technology, Irbid, Jordan; 10 Department of Internal Medicine, Faculty of Medicine, King Abdulaziz University; Inflammatory Bowel Disease Unit, King Abdulaziz University Hospital, Jeddah, Saudi Arabia; 11 Department of Pharmacy Practice, College of Pharmacy, Princess Nourah bint Abdulrahman University, Riyadh, Saudi Arabia; 12 Department of Pharmacy Practice, College of Pharmacy, King Saud bin Abdulaziz University for Health Sciences, Riyadh, Saudi Arabia; 13 Health Technology Assessment Unit, King Fahd Armed Forces Hospital, Jeddah, Saudi Arabia; Mass Eye and Ear: Massachusetts Eye and Ear Infirmary, UNITED STATES OF AMERICA

## Abstract

**Background:**

The Saudi Food and Drug Authority (SFDA) has approved the subcutaneous (SC) administration of infliximab, presenting a more convenient alternative with reduced outpatient visits and diminished expenses compared to the intravenous (IV) administration. However, the financial implications of this formulation have not been examined from the perspective of Saudi payers.

**Methods and materials:**

A prevalence-based budget impact model was developed to evaluate the financial effects of introducing "environment without" versus "with infliximab SC." The model’s time horizon spanned over 2 years (2021–2023), aligning with the biennial national pharmaceutical procurement cycle. The comparison focused on infliximab SC versus all available formulations of infliximab IV in the Saudi market for two inflammatory bowel diseases (IBD): Ulcerative Colitis (UC) and Crohn’s Disease (CD). Treatment comparators’ comparability and dose escalations were substantiated by published studies, utilizing dosing information from the summary of product characteristics. Drug acquisition costs were derived from SFDA registered prices, with IV formulation administration costs included. Scenario analysis assessed the budget impact of infliximab SC introduction at uptake rates ranging from 0% to 100%.

**Results:**

Introducing infliximab SC demonstrated cost-saving potential in the treatment of IBD. At 100% uptake with UC patients for 2 years, infliximab SC resulted in savings of -SAR-31.9 million (-SAR29,145 per patient). Similarly, for CD, introducing infliximab SC at 100% uptake over 2 years yielded savings of -SAR106.2 million (-SAR36,585 per patient).

**Conclusion:**

This study reveals that infliximab SC is associated with cost-saving potential when compared to infliximab IV formulations available in Saudi Arabia. Future research should address uncertainties related to real-world comparative effectiveness, the convenience of administration, patient tolerability, and physician acceptance of the SC formulation of infliximab, alongside comparisons with other TNF-alpha inhibitors.

## Introduction

Tumor necrosis factor (TNF) alpha inhibitors are crucial in treating various inflammatory diseases [1]. The US Food and Drug Administration (FDA) has approved five of these inhibitors–infliximab, adalimumab, etanercept, golimumab, and certolizumab–for many indications [2]. These inhibitors can be used alone or combined with other agents like prednisone, methotrexate, hydroxychloroquine, leflunomide, or sulfasalazine [2].

Before a subcutaneously delivered biosimilar version of infliximab became widely available internationally, the original form and its biosimilars were administered intravenously. Currently, all TNF-alpha inhibitors are given subcutaneously. The infliximab subcutaneous (SC) was initially approved by the European Medicine Agency (EMA) in 2019 [3]; followed by FDA acceptance of phase-III LIBERTY-UC trial submissions in 2023 [4]. This SC formulation, the first of its kind, is administered regardless of body weight in all approved indications [5]. It offers potential benefits in terms of convenience for patients and physicians, reducing outpatient visits and intravenous administration costs [5]. Moreover, it has demonstrated noninferiority to the intravenous formulations in efficacy, safety, and immunogenicity [6].

Comparing Saudi Arabia to other Middle Eastern and North African nations, Saudi Arabia had the highest biosimilar approval rate [7]. The Saudi Food and Drug Authority (SFDA) has authorized 14 TNF-alpha inhibitors, with different administration methods [7]. In a recent budget impact analysis conducted in 2022 for inflammatory bowel disease treatment, infliximab SC showed significant cost savings in the United Kingdom, Germany, France, and Italy but not in Spain. Despite SFDA approval in 2022, a budget impact analysis for Saudi Arabia has not been conducted [8].

In general, because the costs of biologic originators and biosimilars continue to have an impact on the national economy of any country, an economic study should be done before introducing any new version of these TNF-alpha inhibitors to inform formulary and payer decisions. In a recent budget impact analysis of infliximab SC for treating inflammatory bowel disease (IBD), conducted in 2022, the infliximab SC showed significant cost savings in four European countries: United Kingdom, Germany, France, and Italy; but not in Spain^3^. Despite the SFDA’s approval of infliximab SC in 2022, the budget impact analysis of this drug has not yet been carried out in Saudi Arabia [8].

Given the global prevalence of inflammatory diseases like inflammatory bowel diseases (IBDs) and psoriasis [9, 10], which have a significant impact on healthcare budgets. Therefore, an economic study should precede the introduction of new TNF-alpha inhibitors to guide formulary and payer decisions. Although these diseases affect millions worldwide, we specifically aim to assess the potential cost savings of infliximab SC compared to all other infliximab IV versions in ulcerative colitis (UC) and Crohn’s disease (CD) from the Saudi payer’s perspective.

## Materials and methods

### Model overview

**Fig 1:**
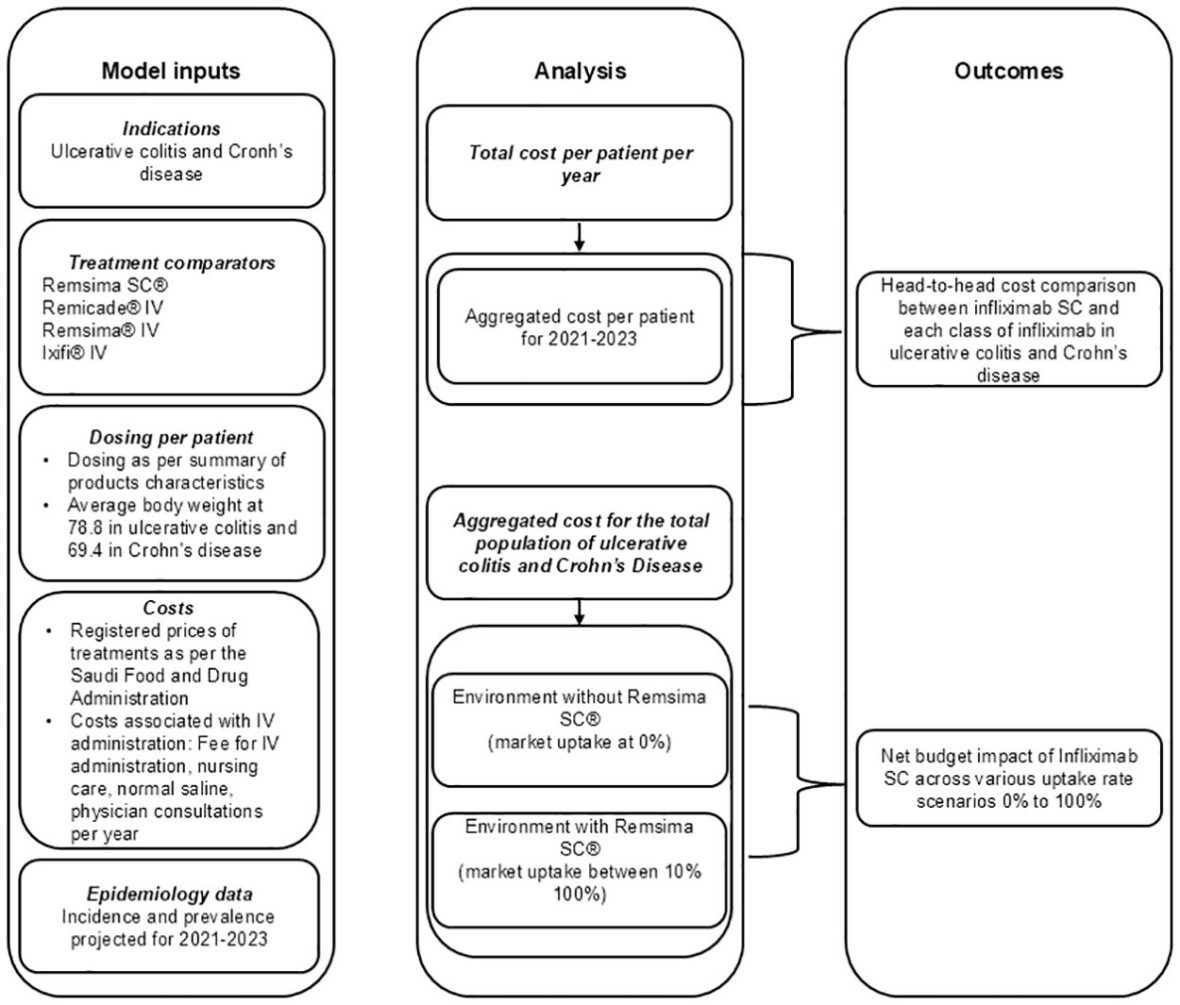
The budget impact model of infliximab formulations for treating inflammatory bowel disease.

As depicted in Fig 1, the budget impact assessment for infliximab SC evaluates its potential application in treating ulcerative colitis (UC) and Crohn’s disease (CD). The comparison involves infliximab SC against all the approved and accessible intravenous (IV) formulations of infliximab in Saudi Arabia. This assessment incorporates evidence on the comparative effectiveness of infliximab SC and infliximab IV in the specified indications, as supported by relevant studies [11–13]. Additionally, it considers the drug acquisition cost based on data from SFDA, and the administration cost associated with IV infliximab formulations in public hospitals [8, 14].

We conducted a prevalence-based budget impact model spanning a 2-year timeframe (2021 to 2023) for assessing the financial implications of introducing infliximab SC in Saudi Arabia. The prevalence data for each indication in the country were obtained from the global database [15]. The choice of a 2-year horizon aligns with Saudi national policy, which involves purchasing pharmaceuticals every two years through the National Unified Procurement Company (NUPCO).

In this model, the total cost per patient per year was calculated in the two indications. This cost was then aggregated to consider the two-year time horizon. Further, the 2-year cost per patient was multiplied by the incidence and the prevalence of ulcerative colitis (UC) and Crohn’s disease (CD) for the two consecutive years 2021–2023.

In this model, we considered two scenarios: one without infliximab SC and one with infliximab SC. The cost per patient was initially computed in each scenario, taking into account the number of patients with specific inflammatory bowel diseases (IBD), the proportion of eligible patients for biological treatment, and the subset of those eligible for infliximab. The net budget impact was then determined by calculating the difference between the scenario with infliximab SC and the scenario without it.

The analysis considers the SFDA prices of the approved infliximab formulations, as outlined in Table 1. The dosing of all infliximab versions was based on summary of products characteristics (SmPC). The dosing for all formulations of infliximab was determined based on the summary of product characteristics (SmPC). Detailed dosing information for various infliximab versions can be found in the supplementary material S1 File. All cost-related findings in this study are reported in Saudi Riyal (SAR), with an exchange rate of 1 SAR equivalent to US $0.27.

**Table 1:** Drug acquisition cost of TNF-alpha inhibitors in Saudi Arabia*.

Brand	Molecule/active ingredient	Formulation	Units per pack	mg per unit	mg per pack	Price per unit (SAR)
Remsima SC^®^	Infliximab	Pre-filled syringe, or Needle, or Auto inject, SC	2	120	240	1,481.50
Remicade^®^	Infliximab	Powder for concentrate for solution for infusion vials, IV	1	100	100	1,650.00
Remsima^®^	Infliximab	Powder for concentrate for solution for infusion vials, IV	1	100	100	1,357.25
Ixifi^®^	Infliximab	Powder for concentrate for solution for infusion vials, IV	1	100	100	1,527.19

SC: subcutaneous; IV: intravenous

*as published by the Saudi Food and Drug Authority 2023

### Assumptions

In this assessment, few assumptions were made. The first assumption was that there was no clinical superiority between infliximab SC and infliximab IV. This assertion relies on findings from a multicenter cohort study conducted by Smith [13]. The second assumption posits that patients consistently adhere to the same treatment throughout the entire evaluation period, without any instances of discontinuation or switching. This assumption is made due to the absence of available data on switching patterns across all indications in Saudi Arabia. The second assumption was that the patient remained on the same treatment for the entire time horizon, with no discontinuation or switching. This is because the switching patterns in all indications are lacking in Saudi Arabia.

### Study population and treatment protocols

As outlined in Table 2, the population figures for Saudi Arabia and prevalence data concerning ulcerative colitis (UC) and Crohn’s disease (CD) were obtained from Global data^15^. The prevalence rates for UC and CD were 0.03% and 0.02%, respectively. These prevalence estimates remained constant from 2013 to 2017 and were incorporated into the model due to the unavailability of data for the years 2021–2023. In Saudi Arabia, the eligibility of patients for infliximab IV treatment stood at 11.24% for UC and 36.82% for CD [14].

**Table 2:** Input data for budget impact analysis (BIA) of Infliximab use in Crohn’s disease and ulcerative colitis.

Inputs	Mean (SD)	Reference
**Epidemiology data**		
**Population in Saudi Arabia 2023**	36,947,025	15
**Prevalence of Ulcerative colitis**	0.03%ȶ	15
**Prevalence of Crohn’s disease**	0.02%ȶ	15
**Patient eligibility for infliximab IV**	Ulcerative colitis 11.24% Crohn’s disease 36.82%	14
**Body weight per indication**		
**Ulcerative colitis**	78.8 (18.4)	20
**Crohn’s disease**	69.4 (15.8)	21
**Rates of dose escalations of infliximab**		
**Ulcerative colitis**		
**Infliximab**	Year 1: 31.8% Year 2: 8.5%	11
**Crohn’s disease**		
**Infliximab**	Year 1: 38% Year 2: 15%	12
**Services and costs** *	Unit cost (SAR)	
**Costs associated with IV administration**		
**Fee for IV administration**	SAR 200	14
**Nursing care**	SAR 50	14
**Normal saline and other meds**	SAR 13	14
**Physician consultation at each visit**	SAR 100	14
**Total**	SAR 363	14
**Costs associated with SC formulation per year**		
**Number of annual visits**	3	14
**Total (SAR 100 per visit)**	SAR 300	14

IV: intravenous; SC: subcutaneous

*All patients on biological treatments were assumed to have the following tests at baseline: Tuberculin test, x-ray, human immunodeficiency virus serology, hepatitis B & C serology, varicella zoster virus serology, herpes simplex virus serology, complete blood count, liver function test, erythrocyte sedimentation rate test, serum albumin, and c-reactive protein test.

Regarding the treatment protocol, for infliximab SC, patients underwent an induction with an intravenous (IV) loading dose of infliximab six weeks prior. This aligns with the clinical trial phase III protocol and prevalent practices documented in the literature [3, 4]. The body weight of patients in each indication was determined based on published studies that provided the average and standard deviation (SD) for a typical patient in each included indication [16–19]. The average body weight for each indication is presented in Table 2. Additionally, the study incorporates dose escalation rates for all infliximab patients in ulcerative colitis (UC) and Crohn’s disease (CD). These rates of dose escalation were derived from published systematic reviews and meta-analyses, estimating escalation rates in the first year of use and subsequent years [11, 12].

The cost of services associated with the route of administration is detailed in Table 2. These costs are reimbursed by the Saudi Ministry of Health as the primary payer. Patients undergoing their initial biologic treatments usually require 13 baseline laboratory tests, including the Tuberculin test, x-ray, human immunodeficiency virus serology, hepatitis B & C serology, varicella-zoster virus serology, herpes simplex virus serology, complete blood count, liver function test, erythrocyte sedimentation rate test, serum albumin, and C-reactive protein test^9^. Individuals receiving intravenous (IV) infliximab typically incur costs for IV technical support, nursing care, normal saline, and physician consultations. On the other hand, patients on subcutaneous (SC) biologics necessitate three annual outpatient visits without any additional services.

### Analyses

First, a direct cost per patient comparison was undertaken, categorizing all infliximab versions based on the Saudi Food and Drug Authority’s (SFDA) classification of originators and biosimilars. The original infliximab (o) is Remicade, while the biosimilar infliximab (b) encompasses Remsima IV and Ixifi. In each treatment comparator and for each indication, the total cost per patient per year was quantified and then aggregated for the two-year time horizon (2021–2023). Subsequently, two-year cost differences between infliximab SC and the IV formulations in ulcerative colitis and Crohn’s disease was Identified.

Second, aggregated population cost was estimated. In this estimation, the cost per patient per year was multiplied by the eligible population of Ulcerative Colitis (UC) and Crohn’s disease (CD) given the projected incidence and the prevalence of ulcerative colitis for the time horizon 2021–2023. This step enabled the model to calculate the aggregated cost for the entire patient population for 2021–2023. Subsequently, a budget impact analysis was carried out over a 2-year span, considering two scenarios: one without Infliximab SC versus an environment incorporating Infliximab SC. A sensitivity analysis covered adoption scenarios ranging from 0% (no Infliximab SC adoption) to 100% (complete replacement with Infliximab SC). In the budget impact analysis, the average cost of infliximab IV formulations, encompassing both originators and biosimilars, was used in the comparison with Infliximab SC, rather than individual brand distinctions. This analysis was executed using Microsoft^®^ Excel^®^ 365 with Visual Basic for Applications (VBA) support.

## Results

**Fig 2:**
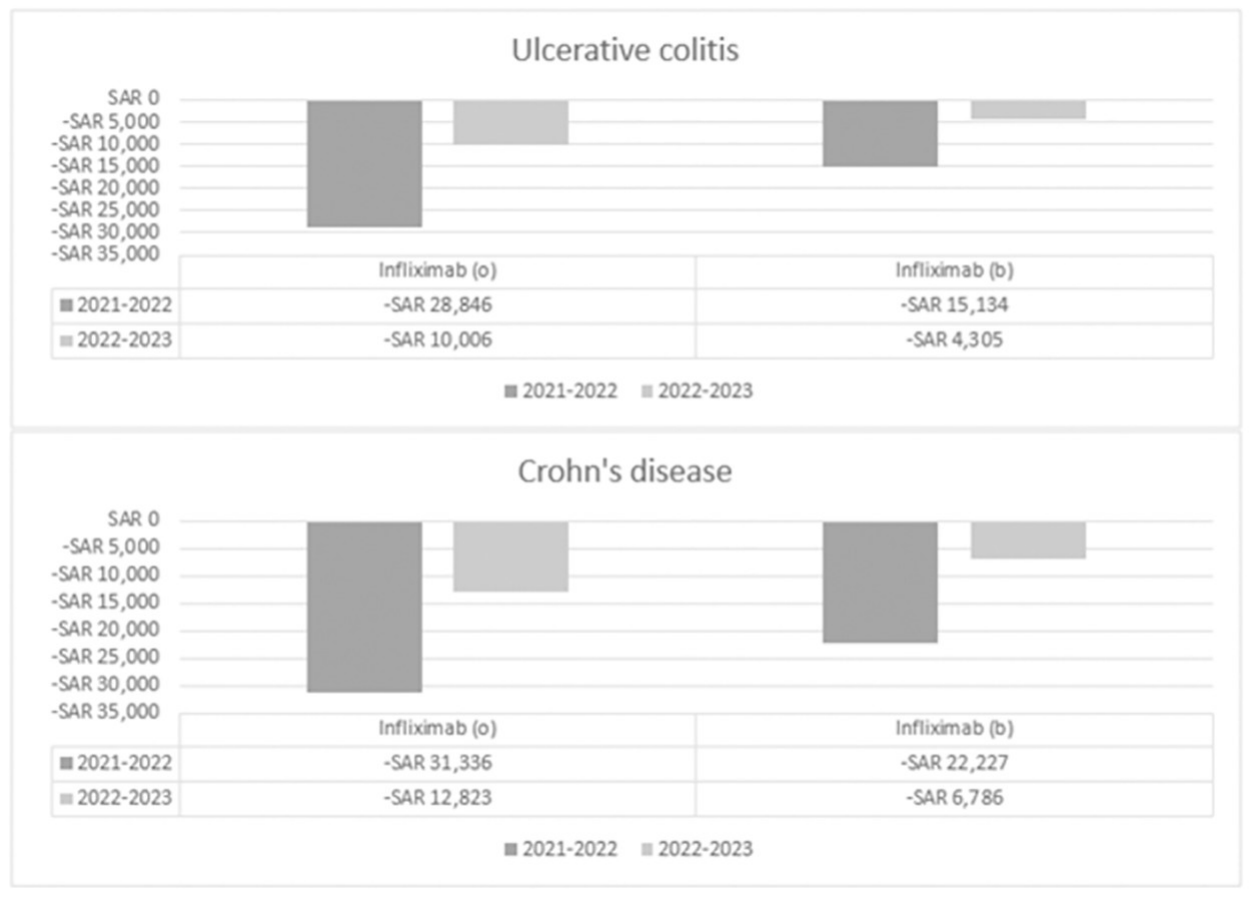
Head-to-head cost comparison between infliximab SC and each class of infliximab in ulcerative colitis and Crohn’s disease.

In ulcerative colitis (UC), the projected total cost of treating a single patient over a 2-year period was estimated at SAR121,947 with infliximab IV (originator), SAR102,534 with infliximab IV (biosimilar), and SAR83,095 with infliximab SC. A breakdown of the annual costs can be found in supplementary material in S2 File. For Crohn’s disease (CD), the total cost of treating one patient over a 2-year span was approximated at SAR127,254 with infliximab IV (originator), SAR112,108 with infliximab IV (biosimilar), and SAR83,095 with infliximab SC. Detailed annual cost breakdowns are available in supplementary material in S3 File. Fig 2 illustrates the direct cost comparison between infliximab SC and infliximab IV for each indication. A negative sign in the figures indicates savings per patient per year, while no sign suggests overspending per patient per year. For both indications UC and CD, infliximab SC demonstrated cost-saving potential when compared to both infliximab (originator) and infliximab (biosimilar) over the 2-year period. The most substantial savings with infliximab SC were observed in CD, amounting to -SAR31,336 per patient in 2021–2022 and -SAR12,823 per patient in 2022–2023.

**Fig 3:**
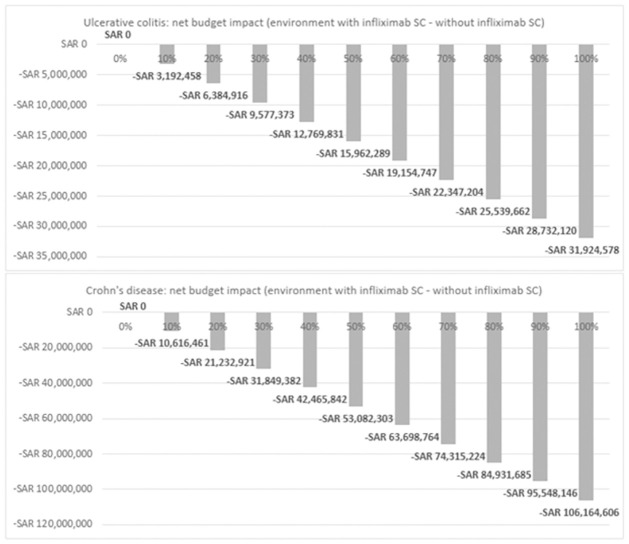
Net budget impact of infliximab SC across various uptake rate scenarios 0% to 100%.

In this evaluation, the total number of patients with UC and CD in Saudi Arabia was 9,745 and 7,881, respectively. Among these, 1,095 UC patients and 2,902 CD patients were eligible for treatment with the infliximab IV formulation. These figures were utilized to project the national budget over a 2-year period in two scenarios: one "without infliximab SC" and the other "with infliximab SC," considering various adoption rates ranging from 0% to 100% for infliximab SC. Fig 3 illustrates the net budget impact between the two scenarios for UC and CD over the 2-year horizon at different infliximab SC uptake rates.

As depicted in Fig 3, the savings associated with infliximab SC reach -SAR31.9 million in UC and -SAR106.2 million in CD at 100% uptake (full adoption). When these savings are divided by the number of eligible patients receiving infliximab IV formulations, the resulting savings per patient over the 2-year period are -SAR29,145 for UC patients and -SAR36,585 for CD patients.

## Discussion

To the best of our knowledge, this is the inaugural economic study examining the economic implications of infliximab SC in contrast to other infliximab IV alternatives, specifically from the viewpoint of the Saudi Arabian payer. Our findings indicate that, at the prices set by SFDA, infliximab SC presents a cost-saving alternative in comparison to all currently accessible infliximab IV options for the treatment of UC and CD.

In both UC and CD, infliximab SC emerged as a cost-saving alternative when compared to the included intravenous (IV) formulations of infliximab. Two critical factors contribute to this outcome. Firstly, the registered price by the SFDA for infliximab SC was lower than that of infliximab IV (originator) and one of the two infliximab IV (biosimilar) options. It is important to note that these findings are contingent upon the current SFDA prices and are susceptible to change over time. Furthermore, the SFDA price points mirror the registered prices of each infliximab version in the Saudi market for out-of-pocket and private payers. However, for the central payer system in Saudi Arabia, the National Unified Procurement Company (NUPCO) prices are typically adopted to represent the price point at which the public health sector acquires pharmaceutical products [20]. Unfortunately, we were unable to conduct the analysis based on NUPCO prices due to their confidential nature. It is noteworthy that NUPCO prices are generally lower than SFDA prices, with an average reduction of approximately 30%, reflecting the volume-based purchasing agreements of NUPCO. In Saudi Arabia, the SFDA plays a pivotal role in regulating drug prices, and the prices determined by the SFDA serve as a benchmark for the out-of-pocket and private payer segments of the healthcare system.

It is important to consider the central payer system in Saudi Arabia, where the NUPCO^®^ is a key entity in acquiring pharmaceutical products for the public health sector [20]. However, due to the confidential nature of NUPCO prices, our assessment primarily relies on the SFDA-registered prices. In a comprehensive analysis, incorporating NUPCO prices would provide a more nuanced understanding of the economic landscape, particularly for the public health sector. Research indicates that NUPCO prices are typically lower than SFDA prices, with an average reduction of around 30%. This price disparity is attributed to the volume-based purchasing agreements facilitated by NUPCO, emphasizing the importance of considering different pricing structures in assessing the economic impact of pharmaceutical options. Unfortunately, due to the dynamic nature of the pharmaceutical market and the confidential nature of certain pricing information, providing exact references for this evolving context proves challenging. However, insights from reputable sources like economic analyses, government reports, and scholarly studies on drug pricing mechanisms in Saudi Arabia would serve as valuable references for further exploration and understanding.

Secondly, the potential for dose escalation of infliximab IV was considered in UC and CD, based on findings from two systematic reviews and meta-analyses that estimated the frequency of dose escalation in these specific indications [11, 12]. Notably, infliximab SC did not undergo dose escalation, as it is not weight-dependent like its intravenous counterpart, infliximab IV. While we acknowledge the significance of dose escalation as a critical practice among healthcare practitioners, our analysis demonstrated that even without implementing dose escalation in UC and CD, infliximab SC still yielded cost savings in comparison to both the originators and biosimilars of infliximab IV. This financial advantage persists because the registered price of infliximab SC remains lower than that of the majority of infliximab IV versions, as outlined by the SDFA.

The findings of this study align with the conclusions drawn from an epidemiology-based budget impact analysis conducted across five European countries: the United Kingdom, Germany, France, Italy, and Spain, specifically focusing on ulcerative colitis (UC) and Crohn’s disease (CD) indications [3]. According to the study, the introduction of infliximab SC for CD patients led to cost savings of €42.0 million in the UK, €59.4 million in Germany, and €46.4 million in France and Italy over a 5-year period. However, it resulted in increased budget expenditure in Spain by €3.8 million. Similarly, for UC patients, the study demonstrated total cost savings of €42.7 million in the United Kingdom, €44.9 million in Germany, €44.3 million in France, and €53.0 million in Italy over the same 5-year duration, with no estimated savings in Spain [3].

In our study, the incorporation of infliximab SC into the treatment of inflammatory bowel disease (IBD) resulted in significant cost savings. A prior study examining the utilization of biologic and non-biologic drugs in IBD treatment reported that infliximab IV was utilized by 11.24% of patients with UC and 36.82% of patients with CD [14]. By adopting these utilization estimates in our analysis, it was determined that 1,095 UC patients and 2,902 CD patients were eligible for treatment with infliximab IV. Consequently, at 100% uptake (full adoption) of infliximab SC, the accrued savings amounted to -SAR31.9 million for UC and -SAR106.2 million for CD. These substantial savings present an opportunity to reallocate resources, potentially increasing access to novel biological treatments for a greater number of IBD patients.

This study holds significance on two fronts. Firstly, it addresses the critical aspect of timing in evidence generation. The recent approval of Infliximab SC by the SFDA marks a pivotal development, and the formulation is anticipated to be integrated into the NUPCO’s plans for 2024–2026. Notably, prior to this study, no economic evidence had been published, making this research timely and crucial in providing insights into the economic impact of Infliximab SC.

Secondly, the study plays a pivotal role in informing decision-making processes regarding the treatment of inflammatory diseases. Notably, professional associations such as the Saudi Gastroenterologist Association (SGA) have released statements on the use of TNF-alpha inhibitors for the treatment of IBD [21]. It’s noteworthy that at the time of the SGA statement, Infliximab SC was not yet available in the Saudi market [21]. With the findings of this study, decision-makers, including healthcare providers and policymakers, can strategically plan for updates to treatment protocols, coverage policies, and resource allocation, aligning with the introduction of Infliximab SC into the market. This ensures that decisions are evidence-based and consider the economic implications for the healthcare system and patients alike.

An additional aspect, not directly measured in this study but crucial for decision-makers to consider, is the convenience associated with administering Infliximab SC compared to the intravenous (IV) formulation. The results of this study might underestimate the true economic value of Infliximab SC, given that patients can self-administer at home, eliminating the need for admission to an inpatient or outpatient clinic. This aspect is particularly significant as it not only enhances the patient experience but also has broader implications for healthcare providers. The convenience of Infliximab SC introduces efficiencies from a provider perspective. The time traditionally spent by nurses or technicians during IV infusions in outpatient wards can be redirected to serve more patients across different areas when an SC formulation replaces an IV formulation. This not only optimizes resource utilization but also contributes to improving overall healthcare service delivery. Thus, decision-makers should recognize and account for these non-measurable yet impactful aspects when evaluating the comprehensive value proposition of Infliximab SC in the context of patient care and healthcare resource management [3].

Our study is subject to certain limitations. While originators such as adalimumab, certolizumab, etanercept, and golimumab are available in Saudi Arabia, they were excluded from the analysis due to the absence of comparative efficacy data against infliximab SC. This lack of data restricts our ability to comprehensively evaluate these treatments in relation to infliximab SC. Additionally, the model employed in our study does not incorporate switching practices. This limitation arises from the absence of real-world data on patterns of switching between different versions of infliximab in Saudi Arabia. The dynamics of treatment switching are complex and can significantly impact economic outcomes, but the current analysis does not account for this aspect. Furthermore, the study does not assess productivity costs. This omission is attributed to the unavailability of data required to estimate such costs. While productivity costs are a relevant factor in the economic evaluation of healthcare interventions, their absence in our study reflects a data constraint. These limitations underscore the need for ongoing research and real-world data collection to enhance the precision and comprehensiveness of economic evaluations in the context of inflammatory bowel disease treatments in Saudi Arabia.

It is crucial to highlight that the clinical positioning of TNF-alpha inhibitors within clinical protocols varies across the studied indications. This variability is particularly evident in patients with co-morbidities, where TNF-alpha inhibitors differ in terms of response rates, adverse events, and tolerability factors [22–24]. When selecting a TNF-alpha inhibitor, these considerations become pivotal, and decision-makers should weigh these factors to determine the most suitable option based on individual patient characteristics and the specific clinical context.

For instance, in the context of IBD, current recommendations favor infliximab as the primary choice, especially in progressive or refractory stages [25, 26]. On the other hand, in the treatment of psoriasis, etanercept monotherapy is recommended as the first choice for patients with moderate to severe conditions [22]. These nuanced differences in clinical guidelines underscore the importance of tailoring treatment decisions to the specific needs and conditions of patients, acknowledging that not all TNF-alpha inhibitors are interchangeable and that individualized approaches are essential for optimizing therapeutic outcomes.

Moreover, the cost factor significantly influences the selection of TNF-alpha inhibitors in many healthcare systems. These biological treatments pose a considerable financial burden for both patients and payers [11]. The expiration of patents for certain TNF-alpha inhibitors has paved the way for the availability of biosimilars, offering substantial cost reductions for patients and payers alike [7, 27–31]. However, the successful adoption of these biosimilars is contingent upon the acceptance of patients and physicians [7, 32]. In addition to cost considerations, the incorporation of innovative technologies, such as artificial intelligence, in predicting the performance of biological treatments is becoming increasingly integral to decision-making in the field [33]. This study aligns with the imperative for payers to evaluate the value of the infliximab SC formulation within the specific context of Saudi jurisdictions. Future assessments should extend their scope to encompass the real-world value of infliximab SC in comparison to all other TNF-alpha inhibitors. These assessments must not only delve into comparative effectiveness but also take into account switching rates between these TNF-alpha inhibitors [34]. By considering these multifaceted factors, healthcare decision-makers can make informed choices that optimize both clinical outcomes and resource utilization.

## Conclusion

In this investigation, infliximab SC demonstrated cost-saving potential in comparison to the available infliximab IV formulations in Saudi Arabia. To enhance our understanding and address existing uncertainties, future studies should delve into real-world comparative effectiveness assessments of infliximab SC against infliximab IV formulations and other TNF-alpha inhibitors. Additionally, aspects such as the convenience of administration, patient tolerability, and physician acceptance to prescribe the subcutaneous formulation of infliximab should be further explored. These endeavors will contribute valuable insights to guide evidence-based decision-making and optimize the utilization of these treatments in clinical practice.

## Supporting information

S1 File Dosing of TNF-alpha inhibitors. (DOCX)

S2 File Total cost per patient per year in ulcerative colitis. (DOCX)

S3 File Total cost per patient per year in Crohn’s disease. (DOCX)
