# Supplementary material for: Budget impact analysis of subcutaneous infliximab (CT-P13 SC) for treating inflammatory bowel disease in Saudi Arabia: Analysis from payer perspective
Source: PLoS One. 2024 Nov 12;19(11):e0312603. doi: 10.1371/journal.pone.0312603 (PMC11556681; doi:10.1371/journal.pone.0312603)
Supplement: S1 File — (DOCX) [file pone.0312603.s001.docx]

**S1: Dosing of TNF-alpha inhibitors:**

**Infliximab IV**

**Severe active ulcerative colitis**

For Adult: Initially 5 mg/kg, then 5 mg/kg after 2 weeks, followed by 5 mg/kg after 4 weeks, then 5 mg/kg every 8 weeks, discontinue if no response 14 weeks after initial dose.

**Severe active Crohn's disease**

For Adult: Initially 5 mg/kg, then 5 mg/kg after 2 weeks, then 5 mg/kg after 4 weeks, if condition has responded, then maintenance 5 mg/kg every 8 weeks.

**Fistulating Crohn's disease**

For Adult: Initially 5 mg/kg, then 5 mg/kg after 2 weeks, followed by 5 mg/kg after 4 weeks, if condition has responded consult product literature for guidance on further doses.

**Infliximab SC**

All indications: the number of injections in year 1 is calculated assuming 1 injection every 2 weeks, starting at week 0 for patients who do not receive an IV loading dose and at week 6 for patients who do receive an IV loading dose.
