# Supplementary material for: Budget impact analysis of subcutaneous infliximab (CT-P13 SC) for treating inflammatory bowel disease in Saudi Arabia: Analysis from payer perspective
Source: PLoS One. 2024 Nov 12;19(11):e0312603. doi: 10.1371/journal.pone.0312603 (PMC11556681; doi:10.1371/journal.pone.0312603)
Supplement: S3 File — (DOCX) [file pone.0312603.s003.docx]

**Table S3:** Total cost per patient per year in Crohn’s disease

| **Crohn’s disease** | | |
| --- | --- | --- |
| **Year**  **Infliximab version** | **2021-2022** | **2022-2023** |
| Infliximab (o) | SAR 76,674 | SAR 50,580 |
| Infliximab (b) | SAR 67,565 | SAR 44,543 |
| Infliximab SC | SAR 46,238 | SAR 38,630 |

Infliximab (o): infliximab originator; Infliximab (b): infliximab biosimilars
